# Induction of Neutralizing Responses against Autologous Virus in Maternal HIV Vaccine Trials

**DOI:** 10.1128/mSphere.00254-20

**Published:** 2020-06-03

**Authors:** Eliza D. Hompe, Jesse F. Mangold, Amit Kumar, Joshua A. Eudailey, Erin McGuire, Barton F. Haynes, M. Anthony Moody, Peter F. Wright, Genevieve G. Fouda, Elena E. Giorgi, Feng Gao, Sallie R. Permar

**Affiliations:** a Duke Human Vaccine Institute, Duke University Medical Center, Durham, North Carolina, USA; b Department of Medicine, Duke University Medical Center, Durham, North Carolina, USA; c Department of Pediatrics, Duke University Medical Center, Durham, North Carolina, USA; d Department of Pediatrics, Dartmouth-Hitchcock Medical Center, Lebanon, New Hampshire, USA; e Los Alamos National Laboratory, Los Alamos, New Mexico, USA; University of Florida

**Keywords:** HIV envelope, autologous virus neutralization, human immunodeficiency virus, maternal vaccination, mother-to-child transmission

## Abstract

A maternal vaccine capable of boosting neutralizing antibody (NAb) responses directed against circulating viruses in HIV-infected pregnant women could effectively decrease mother-to-child transmission of HIV. However, it is not known if an HIV envelope (Env) vaccine administered to infected pregnant women could enhance autologous virus neutralization and thereby reduce this risk of vertical HIV transmission. Here, we assessed autologous virus NAb responses in maternal plasma samples obtained from AIDS Vaccine Evaluation Group (AVEG) protocols 104 and 102, representing historical phase I safety and immunogenicity trials of recombinant HIV Env subunit vaccines administered to HIV-infected pregnant women (ClinicalTrials registration no. NCT00001041). Maternal HIV Env-specific plasma binding and neutralizing antibody responses were characterized before and after vaccination in 15 AVEG 104 (*n* = 10 vaccine recipients, *n* = 5 placebo recipients) and 2 AVEG 102 (*n* = 1 vaccine recipient, *n* = 1 placebo recipient) participants. Single-genome amplification (SGA) was used to obtain HIV *env* gene sequences of autologous maternal viruses for pseudovirus production and neutralization sensitivity testing in pre- and postvaccination plasma of HIV-infected pregnant vaccine recipients (*n* = 6 gp120, *n* = 1 gp160) and placebo recipients (*n* = 3). We detected an increase in Env subunit MN gp120-specific IgG binding in the group of vaccine recipients between the first immunization visit and the last visit at delivery (*P* = 0.027, 2-sided Wilcoxon test). While no difference was observed in the levels of autologous virus neutralization potency between groups, in both groups maternal plasma collected at delivery more effectively neutralized autologous viruses from early pregnancy than late pregnancy. Immunization strategies capable of further enhancing these autologous virus NAb responses in pregnant women will be important to block vertical transmission of HIV.

**IMPORTANCE** Maternal antiretroviral therapy (ART) has effectively reduced but not eliminated the burden of mother-to-child transmission of HIV across the globe, as an estimated 160,000 children were newly infected with HIV in 2018. Thus, additional preventive strategies beyond ART will be required to close the remaining gap and end the pediatric HIV epidemic. A maternal active immunization strategy that synergizes with maternal ART could further reduce infant HIV infections. In this study, we found that two historic HIV Env vaccines did not enhance the ability of HIV-infected pregnant women to neutralize autologous viruses. Therefore, next-generation maternal HIV vaccine candidates must employ alternate approaches to achieve potent neutralizing antibody and perhaps nonneutralizing antibody responses to effectively impede vertical virus transmission. Moreover, these approaches must reflect the broad diversity of HIV strains and widespread availability of ART worldwide.

## Abstract (precis)

Maternal antiretroviral therapy (ART) has effectively reduced but not eliminated the burden of mother-to-child transmission of HIV across the globe, as an estimated 160,000 children were newly infected with HIV in 2018. Thus, additional preventive strategies beyond ART will be required to close the remaining gap and end the pediatric HIV epidemic. A maternal active immunization strategy that synergizes with maternal ART could further reduce infant HIV infections. In this study, we found that two historic HIV Env vaccines did not enhance the ability of HIV-infected pregnant women to neutralize autologous viruses. Therefore, next-generation maternal HIV vaccine candidates must employ alternate approaches to achieve potent neutralizing antibody and perhaps nonneutralizing antibody responses to effectively impede vertical virus transmission. Moreover, these approaches must reflect the broad diversity of HIV strains and widespread availability of ART worldwide.

## INTRODUCTION

Despite widespread efforts to eliminate pediatric HIV infections, mother-to-child transmission (MTCT) of HIV continues to pose a significant global health challenge. With the wide availability of antiretroviral therapy (ART) for HIV-infected women during pregnancy and breastfeeding, as well as for infant prophylaxis, the rate of new HIV infections among infants decreased by 41% from 2010 to 2018 (1). Although approximately 82% of HIV-infected pregnant women across the globe had access to ART in 2018, there were still 160,000 newly acquired pediatric HIV infections in the same year (1). Some of the factors contributing to those new infections were the emergence of drug-resistant HIV strains, late maternal diagnosis or presentation for prenatal care, acute infection during pregnancy or breastfeeding, and poor implementation of ART in resource-limited areas.

In the absence of ART prophylaxis during pregnancy, the MTCT rate is 30 to 40% and can occur antepartum (*in utero*), intrapartum (during labor and delivery), or postpartum (during breastfeeding) (2). Even with optimal implementation of antenatal triple-drug ART, breakthrough transmission can occur, with rates as high as 5% (3, 4). In addition, recent studies have demonstrated that while ART can effectively reduce the rate of MTCT, this reduction comes at the expense of a notable increase in rates of preterm birth and neonatal death, particularly for protease inhibitor-based regimens (4, 5). Moreover, recent reports of increased prevalence of neural tube defects in newborns associated with maternal exposure to dolutegravir-based ART at conception have raised concerns regarding toxicity of ART and highlight an urgent need for additional preventative approaches (6–9). Thus, due to issues of ART access, adherence, incomplete efficacy, and toxicity, further strategies will be required to eliminate MTCT.

Prior studies have indicated that HIV Env-specific antibody (Ab) responses are potentially protective against HIV-1 transmission. The partially effective RV144 vaccine trial of a recombinant gp120 vaccine indicated that vaccine-elicited IgG against variable loops 1 and 2 (V1V2) of gp120 was associated with decreased risk of HIV-1 heterosexual transmission (10–13). While this particular epitope has not been implicated in protection against MTCT, maternal antibodies against both the variable loop 3 (V3) of Env gp120 and the gp41 membrane-proximal external region (MPER) have been shown to correlate with reduced risk of MTCT (14, 15). In addition, studies have demonstrated that heterologous HIV-neutralizing antibodies are found more frequently or in higher titers in nontransmitting than in transmitting mothers (16, 17). However, there are conflicting data regarding the role of maternal antibodies in preventing vertical transmission, as other studies have failed to confirm this association between maternal Env-specific neutralizing antibodies and decreased transmission risk (2, 18). Moreover, some studies have observed the opposite trend, reporting that transmitting women had higher concentrations of maternal IgG against the Env V3 region than nontransmitting women (19). Clearly, further investigation is needed to elucidate the relationship between maternal antibody responses and risk of transmission to the infant.

We previously investigated immune correlates of vertical HIV-1 transmission in a large cohort of HIV clade B-infected U.S. pregnant women from the Women and Infants Transmission Study (WITS) (20). The results demonstrated that maternal IgG against the Env V3 region, maternal plasma neutralization of clade-matched tier 1 but not tier 2 HIV-1 variants, and potency of maternal plasma to block CD4 from binding to clade B HIV-1 Envs each predicted reduced risk of MTCT. Interestingly, these responses were collinear in their prediction of MTCT risk, suggesting that they may be surrogate measures for the same underlying mechanism of virus neutralization that influences infant transmission. In fact, isolated V3-specific monoclonal antibodies (MAbs) that could neutralize tier 1 but not tier 2 heterologous viruses were able to neutralize most autologous viruses isolated from maternal plasma (20). Moreover, it has also been demonstrated that autologous V3 and CD4 binding site (CD4bs) MAbs isolated from chronically HIV-1-infected individuals can neutralize autologous, but not heterologous, tier 2 viruses (21). That non-broadly neutralizing antibodies can potently neutralize autologous circulating viruses is especially pertinent in the unique setting of MTCT, as maternal circulating viruses are the source of the vertically transmitted virus. In a recent study, we also characterized vertically transmitted and nontransmitted maternal HIV Env variants in 16 mother-infant transmitting pairs from the WITS cohort and found that the infant-transmitted virus variants showed significantly greater neutralization resistance to paired maternal plasma than the maternal nontransmitted variants (22). This finding suggests that autologous neutralizing antibody sensitivity may define infant transmitted/founder variants, and therefore, boosting autologous neutralizing antibody responses in HIV-infected pregnant women could be a viable immune-based strategy to decrease vertical transmission.

Yet it is unknown whether vaccination of HIV-infected pregnant women with an Env vaccine would enhance autologous virus-neutralizing antibody responses even transiently. In two historic vaccine trials completed in 1993 to 1995 by the AIDS Vaccine Evaluation Group (AVEG) (protocols 104 and 102), the safety and immunogenicity of recombinant HIV Env gp120 and gp160 were evaluated in HIV-infected pregnant women (23). AVEG 104 participants were randomized 2:1 to receive 300 μg Env subunit MN recombinant gp120 with aluminum phosphate (alum) or placebo control with alum. AVEG 102 participants were randomized 1:1 to receive 640 μg Env subunit LAV recombinant gp160 with alum or placebo control with alum. While the Env vaccines were safe and well tolerated, there was no consistent increase in the maternal immune responses elicited by the vaccines against heterologous viruses in vaccinees compared to placebo recipients (23). Wright et al. reported that two women had measurable autologous virus neutralization on entry to the trial and that two more women, 1 vaccinee and 1 placebo recipient, developed detectable neutralizing antibodies during the course of the trial. Autologous virus neutralization assays were performed with maternal sera and viral isolates cultured from peripheral blood mononuclear cells (PBMCs) at delivery for 6 vaccinees and 3 placebo recipients. Four women had measurable neutralizing antibody to their own strain on completion of immunization, including 3 of 6 (50%) vaccinees and 1 of 3 (33%) placebo recipients.

In the present study, we applied advanced methodologies to more conclusively assess whether immunization of HIV-infected pregnant women with an alum-adjuvanted recombinant Env vaccine would elicit maternal antibody responses that improved autologous virus neutralization responses. We utilized single-genome amplification (SGA) method to characterize representative maternal virus population diversity from pre- and postimmunization time points in 7 vaccinees and 3 placebo recipients and assessed the ability of the corresponding maternal plasma to neutralize these autologous viruses. Additionally, we employed binding antibody multiplex assay (BAMA) to assess maternal plasma IgG binding to a panel of HIV Env antigens. This work offers insights into the feasibility of enhancing maternal autologous virus neutralization and antibody responses through maternal HIV Env vaccination as an adjunctive strategy to protect the infant against HIV-1 acquisition.

## RESULTS

### Env-specific antibody binding responses in vaccinees compared to placebo recipients.

**FIG 1:**
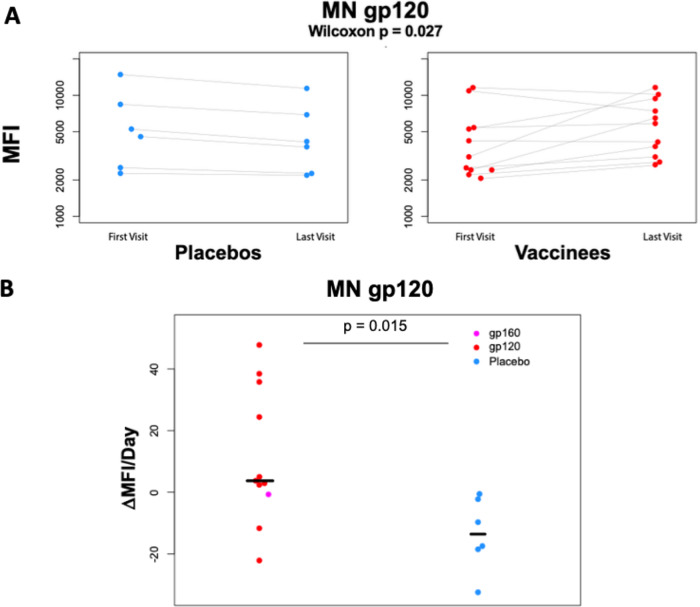
(A and B) Overall and mean change per day in Env subunit MN gp120-specific binding in vaccinees and placebo recipients between first and last visit. (A) Comparison of changes in gp120-specific binding from the first to the last visit between vaccinees (red) and placebo (blue). The between-visits change in gp120-specific binding was statistically significantly higher in vaccinees (*P* = 0.027 by 2-sided Wilcoxon test). Light gray lines link data from the same mother to show the direction of the change between visits. (B) Comparison of mean changes per day in gp120-specific binding per day from the first to the last visit between vaccinees (red or pink) and placebo (blue). Vaccinees had a higher mean gp120-specific binding increase per day than placebo recipients (*P* = 0.015 by 2-sided Wilcoxon test).

**FIG 2:**
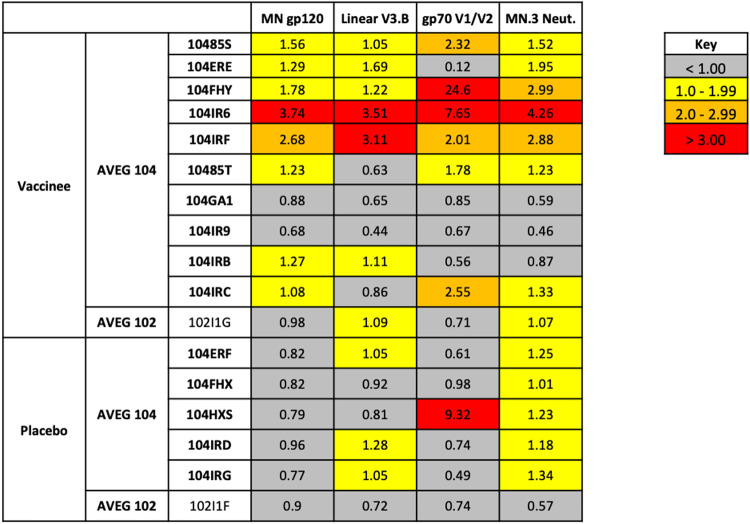
Fold change of antibody response against MN.3 gp120, linear V3.B peptide, and gp70 V1V2 and neutralization response against MN.3 among vaccinees and placebo recipients between the first (visit 1 or 4) and last visit (visit 9).

The magnitude of HIV Env epitope-specific IgG responses prior to and following Env vaccination in HIV-1 infected vaccinated women and placebo recipients was assessed by BAMA. We measured maternal vaccine-elicited responses against clade B MN gp120 protein (Env matched to the vaccine immunogen), gp70 V1V2 protein, and a linear V3.B peptide. Comparing the changes in clade B MN gp120-specific binding responses between the first visit and last visit among study participants, none of the placebo recipients (*n* = 6) showed increase in binding, whereas 8 of 11 vaccinees showed increases in binding over time. Statistically, the overall increase in binding to MN gp120 was significantly higher in vaccinees than in placebo recipients (*P* = 0.027 by Wilcoxon test, Fig. 1A). However, this measure is confounded by the fact that the time intervals between the first and last visits differed greatly across the mothers (range, 38 to 208 days). To account for differences in the timing of visits for each mother, we calculated the mean per day change in MN gp120-specific binding responses between the first visit (visit 1 or 4) and last visit (visit 9). The mean per day increase in clade B MN gp120-specific binding responses between the first and last visits was statistically significantly higher in vaccinees than in placebo recipients (*P* = 0.015 by Wilcoxon test, Fig. 1B). We observed a greater than 3-fold increase in antibody binding responses in 1/11, 2/11, and 2/11 vaccinees against antigens MN gp120, linear V3.B, and gp70 V1V2, respectively (Fig. 2; see also Fig. S1 in the supplemental material). One gp120 vaccinee, 104IR6, demonstrated a greater than 3-fold increase in binding response to all three antigens measured. However, limited changes in antibody binding responses were observed in placebo recipients (Fig. 2; see also Fig. S1). Only 1 of 6 placebo recipients, 104HXS, demonstrated a greater than 3-fold increase in binding response to any antigen (gp70 V1V2) (Fig. 2; see also Fig. S2).

10.1128/mSphere.00254-20.1 FIG S1 HIV Env gp120-, V1V2-, and V3-specific IgG binding and neutralizing Ab responses in HIV-infected pregnant women following immunizations with MN gp120 or gp160. The left *y* axis depicts binding antibody response in log_10_MFI, and gp120 binding responses are indicated by blue lines, V3 responses by red lines, and V1V2 responses by green lines. The right *y* axis depicts neutralization potency, in log_10_ID_50_, and the data are indicated by purple lines. AVEG 102 study participants are indicated in the top row, shaded in gray. Download FIG S1, TIF file, 0.7 MB. Copyright © 2020 Hompe et al. 2020 Hompe et al. This content is distributed under the terms of the Creative Commons Attribution 4.0 International license.

10.1128/mSphere.00254-20.2 FIG S2 Change in antibody response (ΔMFI) against MN.3 gp120, linear V3.B peptide, and gp70 V1V2 and neutralization response (ΔID50) against MN.3 among vaccinees and placebo recipients between the first visit (visit 1 or 4) and last visit (visit 9). Download FIG S2, TIF file, 0.4 MB. Copyright © 2020 Hompe et al. 2020 Hompe et al. This content is distributed under the terms of the Creative Commons Attribution 4.0 International license.

### Autologous virus-neutralizing antibody responses in vaccinees compared to placebo recipients.

**FIG 3:**
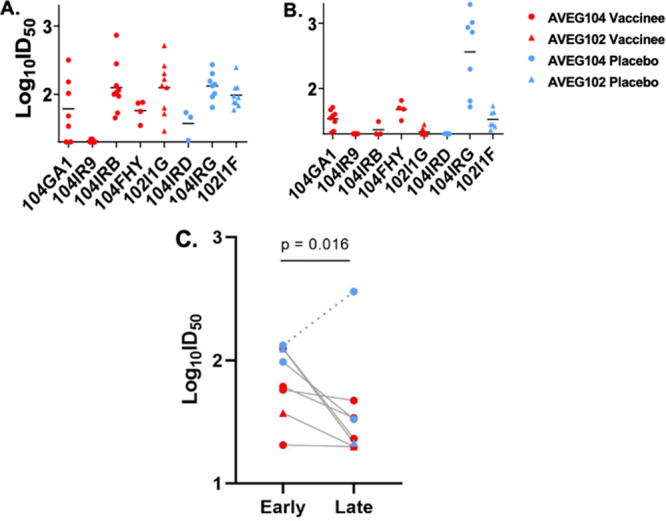
Neutralization of viruses isolated from vaccine and placebo recipient plasma during early and late pregnancy by autologous maternal plasma collected at delivery. (A and B) For each vaccine and placebo recipient, the neutralization potency of maternal plasma at delivery was assessed against the early pregnancy (visits 1 and 4) (A) and late pregnancy (visits 5 to 9) (B) autologous virus populations. The *y* axis depicts neutralization potency in log_10_ID_50_. The *x* axis depicts study participants. AVEG 104 study participants are depicted with circles and AVEG 102 study participants with triangles. Vaccine recipients are shown in red and placebo recipients in blue. Black bars represent geometric means. Early pregnancy plasma autologous viruses were isolated from visit 1, with the exception of visit 4 for mother 104FHY. (C) Geometric means of the neutralization potency of maternal plasma at delivery against autologous viruses from early pregnancy (visits 1 and 4) and late pregnancy (visits 5 to 9) for placebo recipients (blue) and vaccinees (red). The *y* axis depicts neutralization potency in log_10_ID_50_. In both groups, maternal plasma collected at delivery demonstrated greater neutralization potency against early pregnancy autologous viruses than those of late pregnancy, with the exception of one placebo mother, 104IRG (dashed line). This trend became significant after removing the data representing the outlier 104IRG (*P* = 0.016 by 2-sided paired Wilcoxon test).

**FIG 4:**
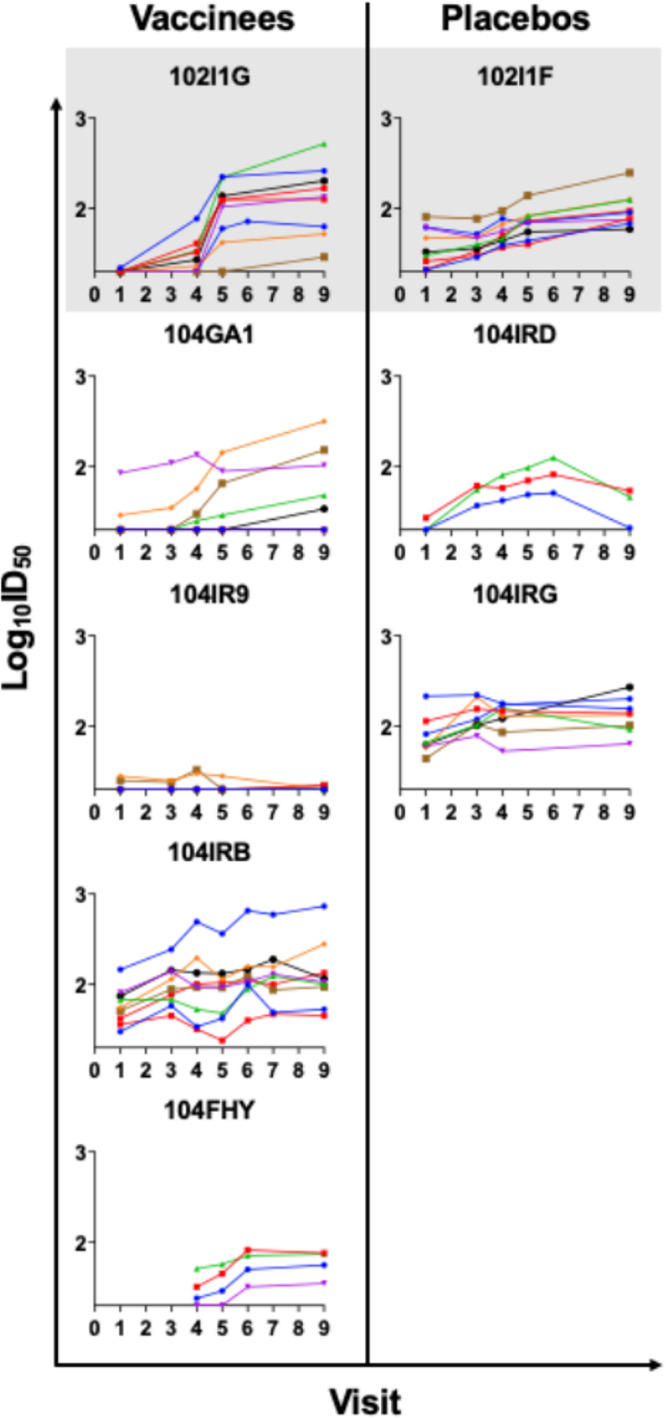
Neutralization potency of autologous maternal plasma against plasma viruses isolated from early pregnancy. Maternal plasma from preimmunization (visit 1), booster visits (visits 3, 4, 5, and 6), and postimmunization (visits 7 and 9) was tested against individual virus variants from visit 1 (except for 104FHY; virus variants are from visit 4). All colored lines represent different viruses. The left *y* axis depicts neutralization potency, in log_10_ID_50_. AVEG 102 study participants are indicated on the top row, shaded in gray.

To determine if gp120 or gp160 vaccination enhanced functional, virus-specific neutralizing antibody responses, we assessed the ability of maternal plasma to neutralize autologous virus variants isolated from plasma collected in early pregnancy before vaccination and in late pregnancy after vaccine boosting. There was no difference between vaccinees and placebo recipients in the ability of maternal plasma collected at delivery to neutralize autologous virus populations isolated from early and late pregnancy visits (Fig. 3A and B; see also Fig. S3). While no significant differences were observed between vaccinees and placebo recipients, in both groups, maternal plasma collected at delivery demonstrated greater neutralization potency against early pregnancy autologous viruses than those of late pregnancy, with the exception of one placebo mother, 104IRG. Comparing the geometric means of the maternal 50% infective dose (ID_50_) values between early and late autologous virus, it was seen that this trend became significant after removing the outlier 104IRG data (*P* = 0.016 by 2-sided paired Wilcoxon test, Fig. 3C). Moreover, the differences in the ability of maternal plasma collected at the first visit (visit 1 and 4) versus last visit (visit 9) to neutralize early pregnancy plasma viruses (visit 1 or 4) were comparable between vaccinees and placebo recipients (Fig. S4). Additionally, we tested the ability of maternal plasma collected from the prevaccination visit (visit 1), the booster visits (visits 3, 4, 5, and 6), and the postvaccination visits (visits 7 and 9) to neutralize autologous virus populations from the prevaccination or early pregnancy visit (visit 1 or 4) (Fig. 4). Taking the results together, there was no significant change in autologous virus neutralization potency over time for vaccinees compared to placebo recipients.

10.1128/mSphere.00254-20.3 FIG S3 Neutralization of viruses isolated from vaccine and placebo recipient plasma during early and late pregnancy by autologous maternal plasma collected at delivery. For each vaccine and placebo recipient, the neutralization potency of maternal plasma at delivery was assessed against the early (visits 1 and 4) and late pregnancy (visits 5 to 9) autologous virus populations. Higher ID_50_ values (darker color) represent greater neutralization potency. Download FIG S3, TIF file, 0.5 MB. Copyright © 2020 Hompe et al. 2020 Hompe et al. This content is distributed under the terms of the Creative Commons Attribution 4.0 International license.

10.1128/mSphere.00254-20.4 FIG S4 Neutralization potency of autologous maternal plasma from visits 1 (preimmunization) and 9 (delivery) against early pregnancy plasma viruses. *y*-axis data depict neutralization potency in log_10_ID_50_. AVEG 104 study participants are indicated with circles and AVEG 102 study participants with triangles. Vaccine recipients are indicated in red and placebo recipients in blue. Early pregnancy plasma autologous viruses were isolated from visit 1, with the exception of visit 4 for mother 104FHY. Download FIG S4, TIF file, 0.2 MB. Copyright © 2020 Hompe et al. 2020 Hompe et al. This content is distributed under the terms of the Creative Commons Attribution 4.0 International license.

### Plasma HIV *env* gene sequence diversity in vaccinees compared to placebo recipients.

**FIG 5:**
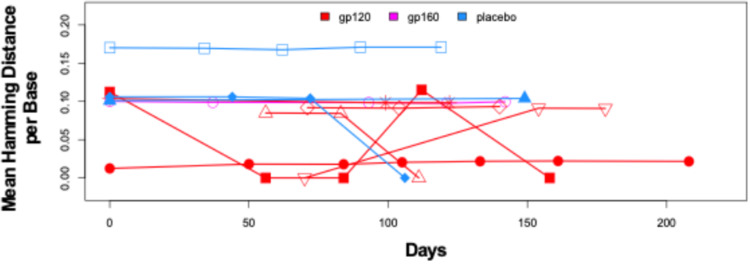
Comparison of change in intersequence Hamming distance per base pair of *env* sequences obtained from vaccinees (*n* = 7) and placebo recipients (*n* = 3) across study visits in days. Each shape represents an individual mother. Red, gp120; pink, gp160; blue, placebo.

Through single genome amplification, we obtained 282 total HIV *env* gene sequences from vaccinees (*n* = 7) and 118 total HIV *env* gene sequences from placebo recipients (*n* = 3) (Table 1). To characterize viral evolution between visits in study participants, we measured viral diversity through determination of the mean pairwise Hamming distance, which was calculated as the number of mutations between all pairs of *env* sequences isolated from one sample divided by the total number of bases in the alignment. The changes in viral diversity between visits observed in the placebo recipients were not significantly different from those observed in the vaccinees, and yet we observed a trend in which vaccinees consistently demonstrated lower HIV *env* gene sequence diversity than placebo recipients (Fig. 5). However, this trend did not reach significance, potentially due to the limited statistical power associated with the small sample size.

**TABLE 1:** Number of maternal *env* gene sequences isolated and functional pseudoviruses produced from each maternal plasma sample

Study cohort	Maternal identifier	Immunization	Visit	Visit date	No. of maternal *env* gene SGAs	No. of functional PSVs
AVEG 102	102I1G	gp160	1	14 September 1993	31	9
			4	21 October 1993	15	NA^*a*^
			5	16 December 1993	17	NA
			6	13 January 1994	8	NA
			9	3 February 1994	21	11
	102I1F	placebo	1	21 May 1993	15	9
			3	24 June 1993	17	NA
			4	22 July 1993	6	NA
			5	19 August 1993	9	NA
			6	16 September 1993	NA	NA
			9	17 September 1993	21	7

AVEG 104	104ERE	gp120	2	17 June 1993	NA	NA
			4	12 August 1993	12	NA
			5	8 September 1993	11	NA
			6	6 October 1993	1	NA
			9	16 October 1993	NA	NA
	104FHY	gp120	1	18 October 1994	NA	NA
			4	28 December 1994	7	4
			5	30 January 1996	7	NA
			6	7 March 1995	8	4
			9	5 April 1995	NA	NA
	104IR6	gp120	1	23 June 1993	NA	NA
			3	4 August 1993	NA	NA
			4	1 September 1993	1	NA
			5	29 September 1993	NA	NA
			6	27 October 1993	NA	NA
			7	24 November 1993	6	NA
			9	18 December 1993	7	NA
	104GA1	gp120	1	25 October 1993	8	7
			3	30 November 1993	NA	NA
			4	4 January 1994	NA	NA
			5	1 February 1994	22	8
			9	24 February 1994	2	2
	104IR9	gp120	1	20 January 1994	24	7
			3	17 March 1994	1	NA
			4	14 April 1994	1	NA
			5	12 May 1994	11	NA
			9	27 June 1994	NA	8
	104IRB	gp120	1	20 April 1994	10	9
			3	9 June 1994	7	NA
			4	13 July 1994	11	NA
			5	3 August 1994	4	NA
			6	31 August 1994	7	NA
			7	28 September 1994	13	NA
			9	14 November 1994	9	8
	104IRD	placebo	1	18 April 1994	3	3
			3	9 June 1994	NA	NA
			4	7 July 1994	NA	NA
			5	4 August 1994	NA	NA
			6	12 September 1994	NA	NA
			9	14 September 1994	4	3
	104IRG	placebo	1	1 November 1994	12	8
			3	15 December 1994	17	NA
			4	12 January 1995	13	7
			9	15 February 1995	1	NA

a NA, not amplifiable.

## DISCUSSION

In this study, we utilized the historical AVEG 102 and 104 maternal HIV Env vaccine trials (1993 to 1995), which assessed the safety and immunogenicity of alum-adjuvanted gp120 and gp160 subunit vaccines in HIV-infected, pregnant women, to investigate the induction of neutralizing antibodies against autologous circulating viruses. Importantly, while the Env vaccines were reported to be safe and immunogenic, the ability of the vaccines to raise neutralizing responses against maternal circulating autologous viruses was not completely assessed (23). With the development of novel techniques to isolate and analyze single HIV variants from plasma, as well as a more sensitive, bead-based binding antibody detection method, we are now better equipped to address this important issue.

We observed that vaccination with either gp120 or gp160 increased MN gp120-specific binding between the first and last visits. Although enhanced autologous virus neutralization due to vaccination was not observed in the AVEG 102 and 104 study cohorts, both vaccine recipient group maternal plasma and placebo group maternal plasma collected at delivery more potently neutralized early pregnancy autologous viruses than those from late pregnancy. These findings reflect the notion of cyclic coevolution of virus and antibody response, in which the neutralizing antibody response acts as a selection pressure for viral escape variants, consistently observed as a vertical transmission bottleneck in mother-infant pairs (22, 24, 25). Thus, immunization strategies capable of more potent B cell stimulation and antibody neutralization to prevent viral escape must be developed in order to be effective in enhancing the mother’s ability to neutralize her own circulating viruses and effective in blocking vertical transmission of HIV.

A subset of participants, 10 vaccinees and 6 placebo recipients, initiated zidovudine therapy during the trial, following recognition that maternal antiretroviral therapy was effective in decreasing the risk of MTCT of HIV (26). Approximately 50% of subjects in each group, 5 vaccinees and 3 placebo recipients, yielded successful isolation of ∼20 maternal *env* gene sequences at the two targeted time points before and after vaccination. Factors such as low maternal plasma volume, low viral load, poor sample storage, and restricted availability of time points limited the successful isolation of sufficient numbers of single-genome amplicons (SGAs) from all participants for larger analyses with greater statistical power. Nevertheless, these cohorts of HIV-infected pregnant women enrolled in a phase I HIV Env subunit vaccine clinical trial represented a unique opportunity to understand the ability of HIV Env vaccination to enhance neutralizing antibody responses in the setting of MTCT.

There are valuable lessons to be learned and key opportunities for improvement in vaccine design based on our analysis. First, next-generation maternal HIV Env vaccination strategies should be developed within the contemporary context of widespread availability of ART for pregnant women. Consequently, studies or trials of future maternal vaccine regimens aimed at preventing vertical transmission of HIV should model the conditions of antiretroviral therapy and viral suppression during pregnancy.

Second, while the majority of vaccines currently licensed for human use in the United States are formulated with an aluminum-based adjuvant, alternative adjuvant selection may play a critical role in the elicitation of protective humoral responses against HIV transmission (27, 28). Moody et al. demonstrated that the use of a combination of a Toll-like receptor 7 or 8 (TLR7/8) agonist with a TLR9 agonist in a squalene-based adjuvant resulted in enhanced HIV Env-specific antibody responses (29). Moreover, during the 2009 influenza A (H1N1) pandemic, a novel squalene-based AS03 adjuvant was safely used among pregnant women in Norway, opening the door for implementation of novel adjuvants beyond alum in pregnancy (30).

Third, the choice of vaccine immunogen must reflect the broad diversity of HIV strains circulating today, specifically, the clade C and B virus subtypes prevalent in sub-Saharan Africa and in the United States and Europe, respectively. One potential approach to overcome this barrier is to employ a multiclade HIV Env immunogen. Importantly, because of the immunological phenomenon of original antigenic sin, it is possible that immunization with a heterologous Env vaccine may recruit memory immune cells in HIV-infected pregnant women, leading to an enhancement of their autologous virus-neutralizing immune responses.

Finally, future studies may explore the potentially protective role of antibody-mediated effector functions beyond neutralization in reducing the risk of MTCT of HIV. Notably, a previous study by Overbaugh et al. suggested that HIV Env-specific IgG-mediated antibody-dependent cell cytotoxicity (ADCC) activity in breastmilk correlates with reduced risk of postnatal vertical transmission (31). Moreover, we previously demonstrated that passive infusion with a cocktail of nonneutralizing antibodies provided partial protection against postnatal SHIV acquisition in an infant nonhuman primate oral challenge model (32). It is therefore possible that elicitation of the full breadth of the polyfunctional antiviral activity of the humoral immune response, and not only the autologous neutralization response, will be critical for a maternal HIV vaccine to protect against MTCT.

Meanwhile, in the absence of a safe and effective maternal HIV vaccine, promising non-ART-based maternal and/or infant interventions should be pursued. Strategies in development include passive administration of broadly neutralizing antibodies (bNAb) and antibody-like entry inhibitors. Maternal bNAb responses are not protective against MTCT and have even been suggested to be a potential risk factor for MTCT of HIV (33). Notably, in the setting of infection during pregnancy, bNAbs of a single specificity may not offer protection but may instead select for neutralization-resistant, infant transmitted-founder HIV variants (34). However, these data do not preclude administration of a cocktail of bNAbs targeting multiple specificities to mitigate the potential introduction of mutations that enable viral escape from any single bNAb. Similar in intent and yet different in approach to bNAbs, engineered antibody-like entry inhibitor molecules (eCD4-Ig) that simultaneously emulate both the target CD4 receptor and a coreceptor, CXCR4 or CCR5, have demonstrated a capacity for high neutralization breadth and a lower potential for viral escape than a single bNAb (35). In contrast to the concerns associated with the administration of single bNAb as a maternal intervention, a recent phase I study has demonstrated the safety, tolerability, and pharmacokinetics of passive administration of VRC01-LS bNAb in HIV-exposed infants as adjunctive therapy to ART to further reduce postnatal transmission via breastfeeding (36). Thus, while development of a safe and highly effective maternal HIV vaccine remains elusive, passive immunization strategies may be used to complement existing maternal and infant ART regimens.

### Conclusion.

In this study, we assessed the autologous virus neutralization responses of maternal plasma collected at delivery against circulating viruses isolated from early and late pregnancy in HIV-infected women vaccinated with HIV Env subunit recombinant gp120 or gp160 adjuvanted with aluminum phosphate from the historical AVEG 102 and 104 phase I trials. Vaccination of HIV-infected pregnant women with recombinant MN gp120 or gp160 adjuvanted with alum boosted HIV Env-specific antibody binding responses between the first and last visits against the original vaccine antigen, compared to placebo recipients, yet vaccination failed to augment the ability of maternal plasma collected at delivery to neutralize heterologous or autologous viruses between the first and last visits. Moreover, vaccination had no evident impact on maternal viral diversity at delivery. These findings indicate that further optimization of the choice of vaccine immunogen and adjuvant will be necessary to effectively augment autologous virus neutralization responses in HIV-infected pregnant women as a strategy to synergize with ART and reduce MTCT of HIV.

## MATERIALS AND METHODS

### Study subjects.

**FIG 6:**
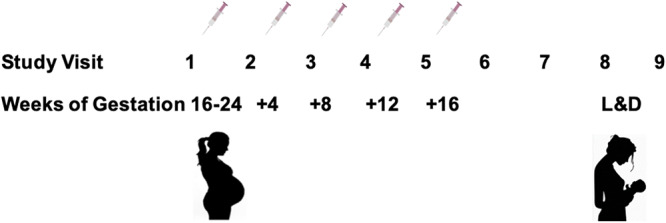
Immunization schedule in AVEG 102/104 studies. Pregnant, HIV-infected women with CD4^+^ T cell counts of >400/mm^3^ were enrolled in the AVEG 102/104 studies. In the AVEG 102 protocol, women received 640 μg of gp160 plus alum (*n* = 1) or placebo (alum plus diluent) (*n* = 1). In the AVEG 104 protocol, women received 300 μg of gp120 plus alum (*n* = 17) or placebo (*n* = 9). The primary immunization was given at visit 2 (between 16 and 24 weeks of gestation). Monthly booster injections were subsequently given 4 weeks apart for the duration of pregnancy (visits 3 to 6) for up to 5 total immunizations (median, 5; range, 4 to 5). Visit 9 was labor and delivery.

Maternal plasma samples were obtained from AIDS Vaccine Evaluation Group (AVEG) protocols 104 and 102, a phase I study of safety and immunogenicity of MN rgp120 and rgp160 HIV-1 vaccines in HIV-infected pregnant women (ClinicalTrials.gov; ClinicalTrials registration no. NCT00001041). In the AVEG 104 protocol, 26 HIV-infected pregnant women with CD4^+^ T cell counts of >400/mm^3^ were enrolled in the second trimester of healthy pregnancy and were randomized to receive either 300 μg of MN rgp120 (Genentech) with alum (*n* = 17) or alum with diluent (*n* = 9) between 16 and 24 weeks of gestation (23). Booster immunizations were administered monthly, until delivery, for a minimum of 3 vaccine doses and a maximum of 5 vaccine doses (Fig. 6). Similarly, 2 HIV-infected pregnant women were enrolled with the same criteria in the AVEG 102 protocol, though these women instead received either 640 μg of LAV rgp160 (VaxSyn, MicroGeneSys) with alum (*n* = 1) or alum with diluent (*n* = 1). Maternal plasma samples from multiple visit time points were available for 15 AVEG 104 participants (*n* = 10 MN rgp120 vaccine, *n* = 5 placebo) and 2 AVEG 102 participants (*n* = 1 LAV rgp160 vaccine, *n* = 1 placebo) (Table 1).

### Ethics statement.

Original study protocols AVEG 102 and 104 were approved by local institutional review boards at the seven sites involved in the original study (23). Informed consent was obtained from all women and also from their partners when available. In the present study, the use of deidentified maternal samples from the AVEG 102 and 104 protocol cohorts was deemed exempt by the Duke University Institutional Review Board. Moreover, in this study, individual patient identification (PTID) numbers are instead represented by PubID numbers.

### Viral RNA extraction and cDNA synthesis.

Viral RNA was extracted from the plasma sample from each mother with a QIAamp Viral RNA minikit (Qiagen) and subjected to reverse transcription for cDNA synthesis using 1× reaction buffer, 0.5 mM (each) deoxynucleoside triphosphate (dNTP), 5 mM dithiothreitol (DTT), 2 U/ml RNaseOUT, 10 U/ml of SuperScript III reverse transcription mix (Invitrogen), and 0.25 mM antisense primer 1.R3.B3R (5′-ACTACTTGAAGCACTCAAGGCAAGCTTTATTG-3′), located in the HIV-1 *nef* open reading frame.

### Single-genome amplification.

Full-length envelope (*env*) genes were then amplified by nested PCR from diluted viral cDNA, as previously described (22, 37). Briefly, cDNA was subjected to endpoint dilution in 96-well plates (Applied Biosystems) with the goal of <30% positive amplification, in order to maximize the likelihood of obtaining a single genome. First-round PCR was carried out with 1× buffer, 2 mM MgSO_4_, 0.2 mM (each) dNTP, 0.2 μM (each) primer, and 0.025 U/μl Platinum *Taq* high-fidelity polymerase (Invitrogen) in a 20-μl reaction mixture. For the first round of PCR amplification, the primer pairs used were Env 5’ex (5′-TAGAGCCCTGGAAGCATCCAGGAAG-3′) and Env 3’ex (5′-TTGCTACTTGTGATTGCTCCATGT-3′), Env 5’ex and 2.R3.B6R (5′-TGAAGCACTCAAGGCAAGCTTTATTGAGGC-3′), or 07For7 (5′-AAATTAYAAAAATTCAAAATTTTCGGGTTTATTACAG-3′) and 2.R3.B6R. The following PCR conditions were used for round 1 amplification: 1 cycle of 94°C for 2 min followed by 35 cycles of 94°C for 15 s, 55°C for 30 s, and 68°C for 3 min 30 s for the Env 5’ex/3’ex primers (4 min 30 s for Env 5’ex/2.R3.B6R and 5 min 30 s for 07For7/2.R3.B6R), followed by a final cycle of 68°C for 10 min. Then, a second round of PCR amplification was carried out with 2 μl of the first-round product as the template, 0.2 μM (each) primer, and the same PCR mixture as that used in round 1, in a 50-μl reaction mixture. The primer pairs used for second-round PCR were Env 5’in (5′-TTAGGCATCTCCTATGGCAGGAAGAAG-3′) and Env 3’in (5′-GTCTCGAGATACTGCTCCCACCC-3′), Env 5’in and 2.R3.B6R (5′-TGAAGCACTCAAGGCAAGCTTTATTGAGGC-3′), or Low2c (5′-TGAGGCTTAAGCAGTGGGTTCC-3′) and VIF1 (5′-GGGTTTATTACAGGGACAGCAGAG-3′). Round 2 conditions were one cycle of 94°C for 2 min followed 35 cycles of 94°C for 15 s, 55°C for 30 s, and 68°C for 3 min 30 s for the Env 5’in/3’in primers (4 min 30 s for Env 5’in/2.R3.B6R and 5 min 30 s for Low2c/VIF1) followed by 1 cycle of 68°C for 10 min. Round 2 PCR amplicons were visualized using precast 1% agarose E-gels (Invitrogen), purified with a AMPure XP magnetic bead purification system (Agencourt), and sequenced for the HIV *env* gene by Sanger sequencing (Table 1). Due to the limited volume of maternal plasma samples available for neutralization testing, we aimed to isolate approximately 20 to 30 maternal *env* gene single-genome amplicons (SGA) at each time point and thereafter selectively produce 8 to 10 functional pseudoviruses from SGAs isolated at the first and last visits for each participant.

### HIV *env* gene genetic analysis.

Sequences were assembled using the Sequencher program (Gene Codes) and manually edited. Chromatograms were examined for sites of ambiguity, or double peaks per base read, and sequences containing multiple base peaks at a single position were marked as such and not studied further. Envelope sequences were aligned using the Gene Cutter tool available in the HIV Sequence Database of the Los Alamos National Laboratory (LANL) website (http://www.hiv.lanl.gov/content/sequence/GENE_CUTTER/cutter.html) and manually edited further in Seaview (Version 4) (38).

### Pseudovirus production and infectivity analysis.

Using a previously described sequence selection algorithm (22), approximately 8 to 10 maternal Env variants were selected from the preimmunization time point (visit 1) and postimmunization time point (visit 9) for Env pseudovirus production. Variants representing major clusters of the phylogenetic trees were selected to represent the full range of *env* genetic diversity in maternal plasma. To produce functional pseudoviruses from the HIV-1 *env* sequences, cytomegalovirus (CMV) promoter was added to the *env* genes by overlapping PCR as previously described (39) and the products were cotransfected with a backbone plasmid lacking the *env* gene (SG3Δenv) in 293T cells (American Tissue Culture Collection, Manassas, VA). 293T cells (approximately 4.5 × 10^6^) were seeded in a T-75 flask (Corning, Corning, NY) containing growth media (GM) (Dulbecco’s modified Eagle’s medium [DMEM]–10% fetal bovine serum [FBS]–1% penicillin-streptomycin containing HEPES; Thermo Fisher, Waltham, MA) and incubated overnight at 37°C and 5% CO_2_. A 4-μg volume of Env DNA containing CMV promoter was combined with 4 μg of SG3Δenv backbone, and FuGene 6 transfection reagent (Roche Diagnostics) was added per the manufacturer’s instructions. The mixture was then added to the T-75 flask, which was incubated at 37°C for 48 h. Supernatant containing pseudovirus was harvested and stored at −80°C with a final 20% concentration of FBS. To measure the infectivity of the pseudoviruses, 20 μl of pseudovirus was added in duplicate to a 96-well flat-bottom plate and then a 100-μl volume of TZM-bl cells (catalog no. 8129; NIH AIDS Reagent Program; from John Kappes and Xiaoyun Wu) was added (10,000 cells/100 μl GM with 10 μg/ml of DEAE-dextran). After a 48-h incubation at 37°C and 5% CO_2_, 100 μl of culture medium was removed and 100 μl of Bright-Glo luciferase (Luc) reagent (Promega) was added. The mixture was incubated for 2 min at 25°C, 100 μl was subsequently transferred to a 96-well black plate, and luminescence was measured immediately on a Victor X3 multilabel plate reader (PerkinElmer).

### TZM-bl neutralization assay.

Neutralization of autologous pseudoviruses by maternal plasma was measured using a Luc reporter gene assay in TZM-bl cells (catalog no. 8129; NIH AIDS Reagent Program; from John Kappes and Xiaoyun Wu), as previously described (40). Before the assay was performed, plasma was heat inactivated by incubation for 30 min at 56°C. Plasma samples were added at a starting dilution of 1:20 and diluted 3-fold serially. Then, the plasma samples were incubated with virus for 1 h at 37°C. TZM-bl cells were added, and the mixture was incubated for 48 h. Luminescence was then measured using Bright-Glo luciferase reagent and a Victor X3 luminometer, and luminescence values used to calculate the ID_50_, or the dilution at which the relative luminescence units (RLU) were reduced by 50% compared to virus control wells. VRC01 was used as a positive control in each experiment, and murine leukemia virus (SVA.MLV) served as a negative control for the assay (41).

### Binding antibody multiplex assay (BAMA).

HIV-1 Env-specific IgG responses of maternal plasma against a panel of HIV-1 antigens were detected using a customized BAMA, as previously described (42). HIV-1 antigens were covalently coupled to carboxylated fluorescent beads (Bio-Rad Laboratories), and IgG binding to the bead-coupled antigens was measured. The antigen panel for IgG BAMAs included biotinylated linear V3 loop peptide V3.B (Bio V3 B) and the following 4 proteins: MNgp120, Gp70 B.MN V3, Gp70 B.CaseA_V1V2, and HIV-1 MN recombinant gp41 (REC MN gp41; ImmunoDiagnostics) (Table S1). The antigen-coupled beads were incubated with diluted plasma samples (1:100 for MNgp120, Gp70 B.MN V3, and Gp70 B.CaseA_V1V2; 1:2,000 for V3.B and REC MN gp41) for 30 min at room temperature (20 to 25°C). HIV Env-specific IgG was then detected with phycoerythrin (PE)-conjugated mouse anti-human IgG (Southern Biotech, Birmingham, AL) at 2 μg/ml. Beads were washed, resuspended, and acquired on a Bio-Plex 200 instrument (Bio-Rad Laboratories). Blank beads were used to account for nonspecific binding, and HIV immunoglobulin (HIVIG) was used as a positive control for all assays. The magnitude of antibody binding to the panel of HIV-1 Env antigens was measured as mean fluorescent intensity (MFI). MFI values for conformational antigens containing gp70 were background-adjusted by subtracting the MFI values determined for gp70 MulV. All MFI values were background-adjusted by subtracting the MFI values of coupled beads without sample. A positive HIV Env-specific antibody response was considered to be represented by an MFI value of >100. The criteria for reporting sample MFI values included ≤20% coefficient of variation with a bead count of ≥100 for each sample. All assays tracked the 50% effective concentration and maximum MFI of the positive-control HIVIG and protein standards CH58, B12, and 7B2 by Levey-Jennings charts to ensure data consistency.

10.1128/mSphere.00254-20.5 TABLE S1 Amino acid sequences for antigens used in AVEG 102/104 plasma binding antibody multiplex assays (BAMA). Download Table S1, DOCX file, 0.01 MB. Copyright © 2020 Hompe et al. 2020 Hompe et al. This content is distributed under the terms of the Creative Commons Attribution 4.0 International license.

### Statistics.

We tested for differences in antibody binding and neutralization responses between placebo and vaccine recipients using 2-sided Wilcoxon tests, comparing the values at the first visit to those at the last visit. Because the time intervals between visits differed across study subjects, we also tested the change per day between visits. All statistical analyses and graphs were produced using R (43).
