# Supplementary figures and images for: Induction of Neutralizing Responses against Autologous Virus in Maternal HIV Vaccine Trials
Source: mSphere. 2020 Jun 3;5(3):e00254-20. doi: 10.1128/mSphere.00254-20 (PMC7273346; doi:10.1128/mSphere.00254-20)

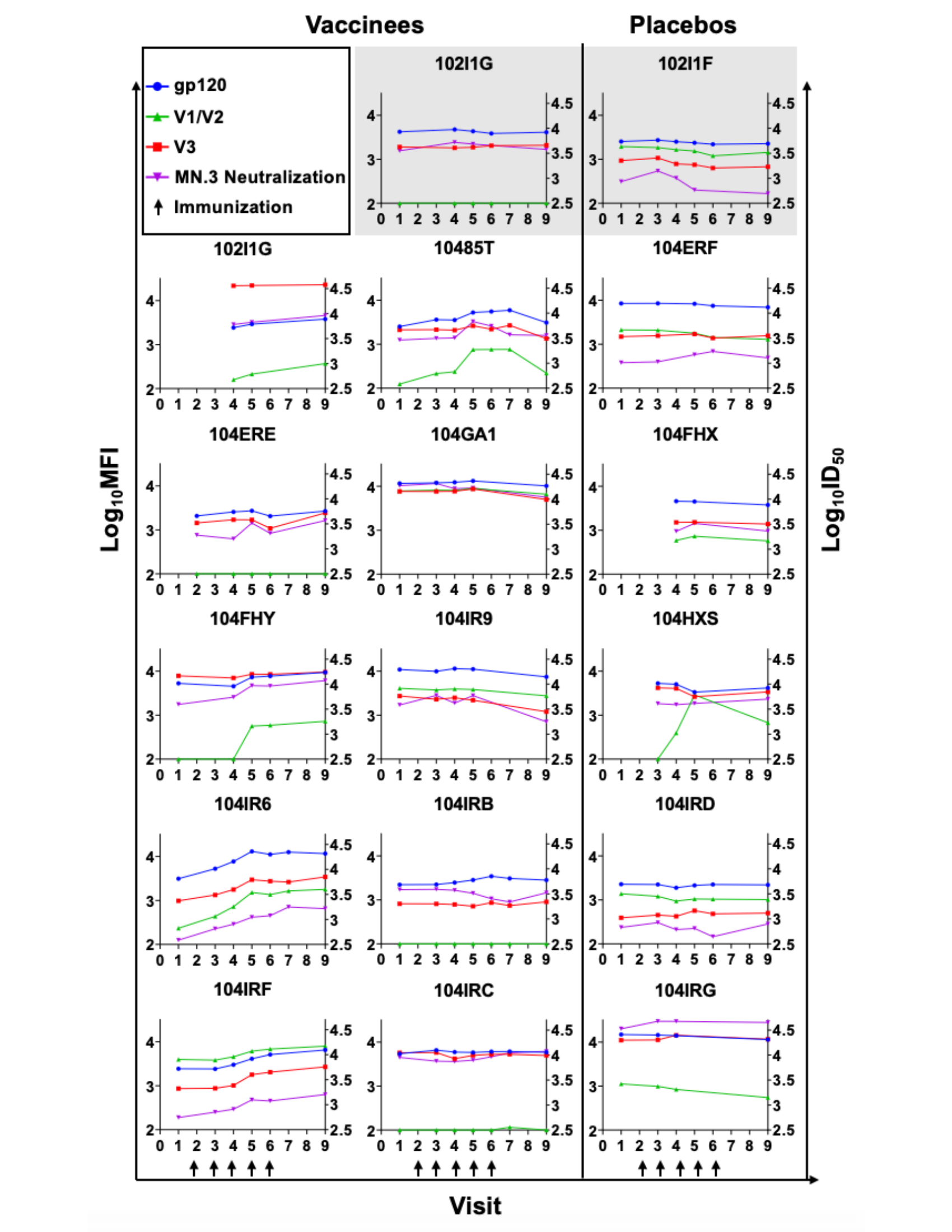

Supplement: FIG S1 [file mSphere.00254-20-sf001.tif]

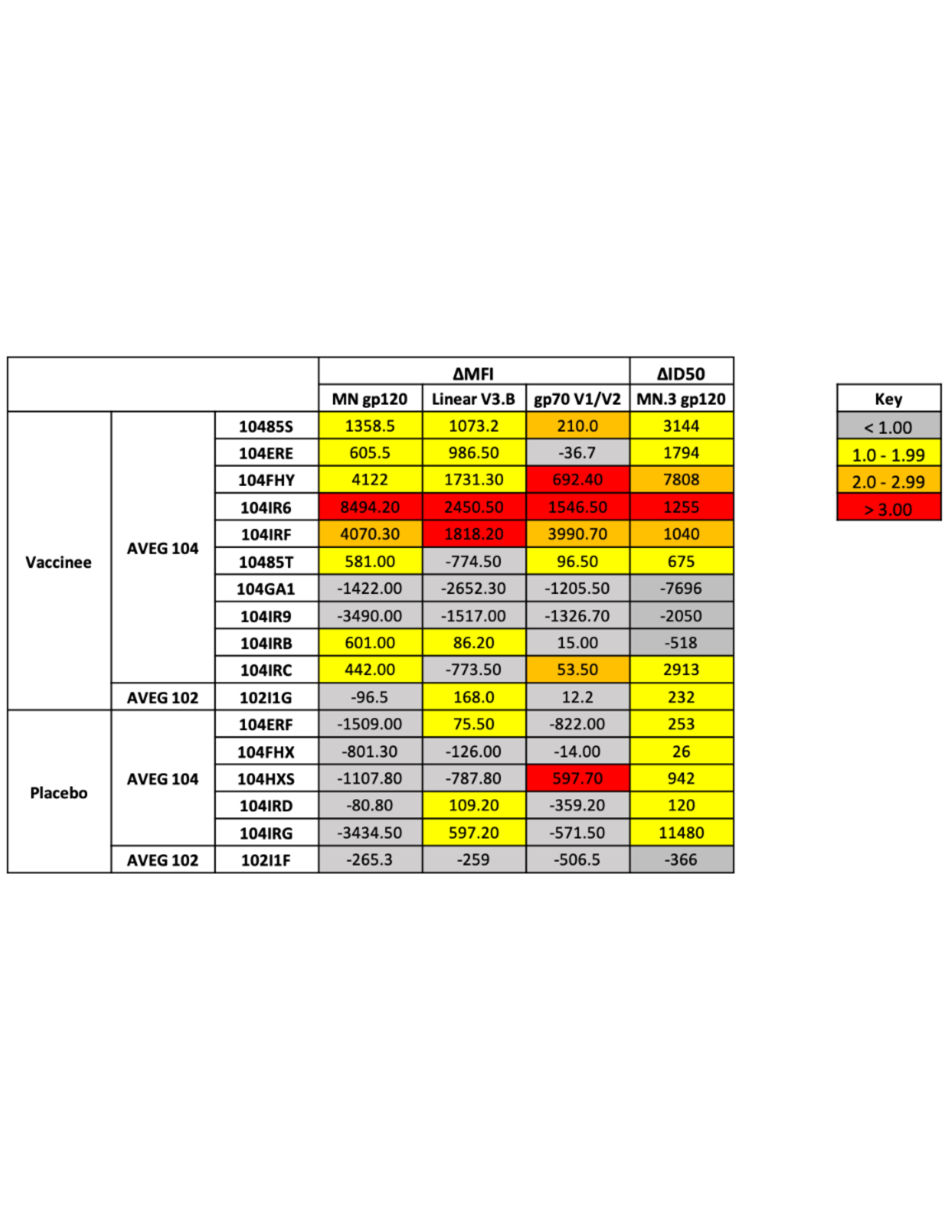

Supplement: FIG S2 [file mSphere.00254-20-sf002.tif]

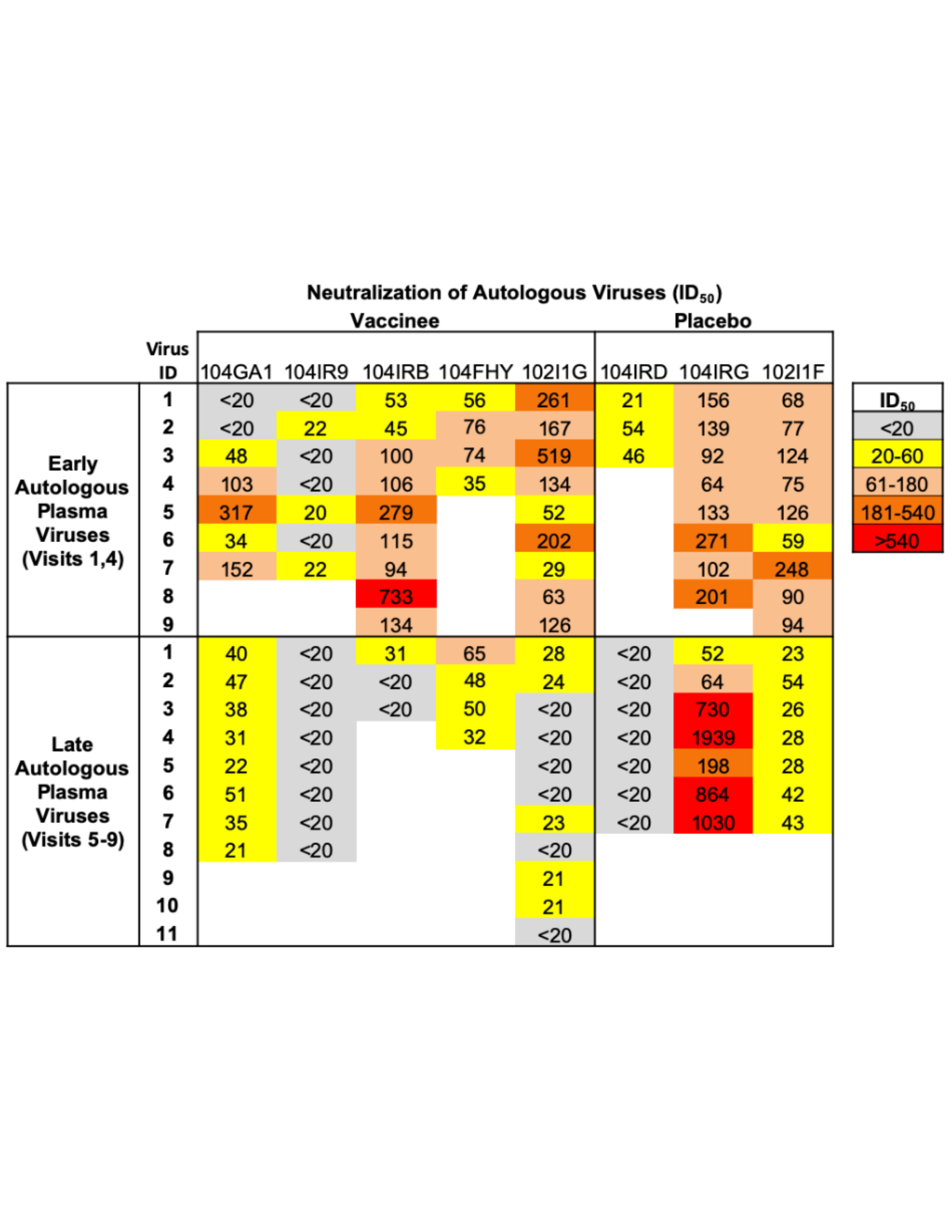

Supplement: FIG S3 [file mSphere.00254-20-sf003.tif]

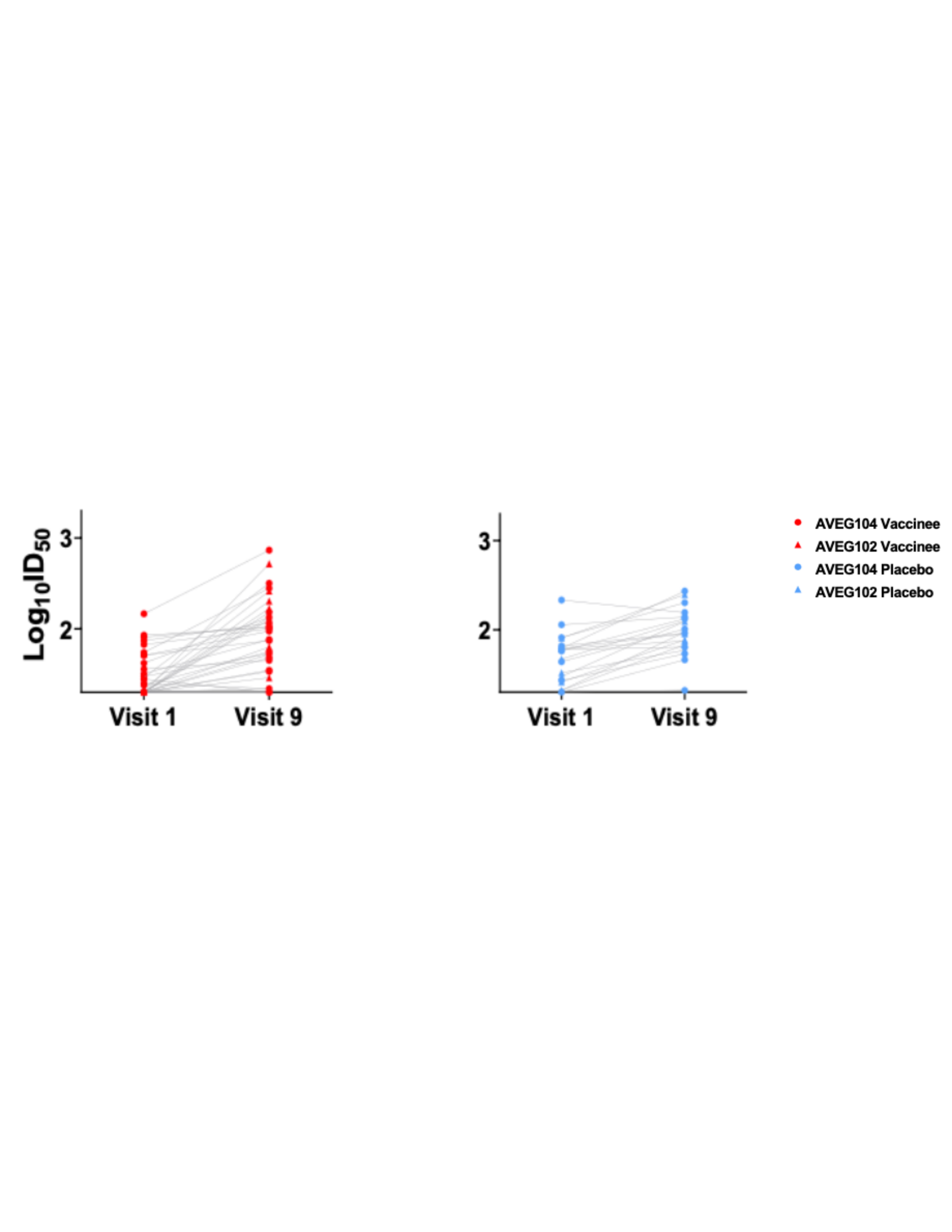

Supplement: FIG S4 [file mSphere.00254-20-sf004.tif]
