# Supplementary material for: Induction of Neutralizing Responses against Autologous Virus in Maternal HIV Vaccine Trials
Source: mSphere. 2020 Jun 3;5(3):e00254-20. doi: 10.1128/mSphere.00254-20 (PMC7273346; doi:10.1128/mSphere.00254-20)
Supplement: TABLE S1 [file mSphere.00254-20-st001.docx]

| Assay | Protein | Peptide | Sequence |
| --- | --- | --- | --- |
| BAMA | MN gp120 |  | TEKLWVTVYYGVPVWKEATTTLFCASDAKAYDTEAHNVWATHACVPTDPNPQEVELVNVTENFNMWKNNMVEQMHEDIISLWDQSLKPCVKLTPLCVTLNCTDLRNTTNTNNSTDNNNSKSEGTIKGGEMKNCSFNITTSIGDKMQKEYALLYKLDIEPIDNDSTSYRLISCNTSVITQACPKISFEPIPIHYCAPAGFAILKCNDKKFSGKGSCKNVSTVQCTHGIRPVVSTQLLLNGSLAEEEVVIRSEDFTDNAKTIIVHLKESVQINCTRPNYNKRKRIHIGPGRAFYTTKNIKGTIRQAHCIISRAKWNDTLRQIVSKLKEQFKNKTIVFNPSSGGDPEIVMHSFNCGGEFFYCNTSPLFNSIWNGNNTWNNTTGSNNNITLQCKIKQIINMWQKVGKAMYAPPIEGQIRCSSNITGLLLTRDGGEDTDTNDTEIFRPGGGDMRDNWRSELYKYKVVTIEPLGVAPTKAKRRVVQREKR |
| BAMA | Rec MN gp41 |  | TVQARLLLSGIVQQQNNLLRAIEAQQNMLQLTVWGIKQLQARVQAVERYLKDQQLLGFWGCSGKLICTTTVPWNASWSNKSLDDIWNNMTWMQWEREIDNYTSLIYSLLEKSQTQQEKNEQELLGLDKWESLWNWFDITNWLENRVRQGYSPLSLQTRPPVPRGPDRPEGIEEEGGERDRDTSGRLVHGFLAIIWVD |
| BAMA |  | Bio-V3.B | NNTRKSIHIGPGRAFYATGDIIGDIRQAHC |
| BAMA |  | gp70 B.MN V3 | TRPNYNKRKRIHIGPGRAFYTTKNIKGTIRQAH |
| BAMA |  | gp70 B.CaseA V1V2 | CIDLRNATNATSNSNTTNTTSSSGGLMMEQGEIKNCSFNITTSIRDKVQKEYALFYKLDIVPIDNPKNSTNYRLISC |
